# Supplementary material for: A Cas-embedding strategy for minimizing off-target effects of DNA base editors
Source: Nat Commun. 2020 Nov 27;11:6073. doi: 10.1038/s41467-020-19690-0 (PMC7695861; doi:10.1038/s41467-020-19690-0)
Supplement: Supplementary file 1 — Supplementary Information [file 41467_2020_19690_MOESM1_ESM.pdf]

Supplementary Information

Liu et al

A Cas-Embedding Strategy for Minimizing Off-Target Effects of DNA Base Editors

Supplementary Figure 1: Genetic screen for tolerant nCas9 sites

Supplementary Figure 2: GOTI data in detail

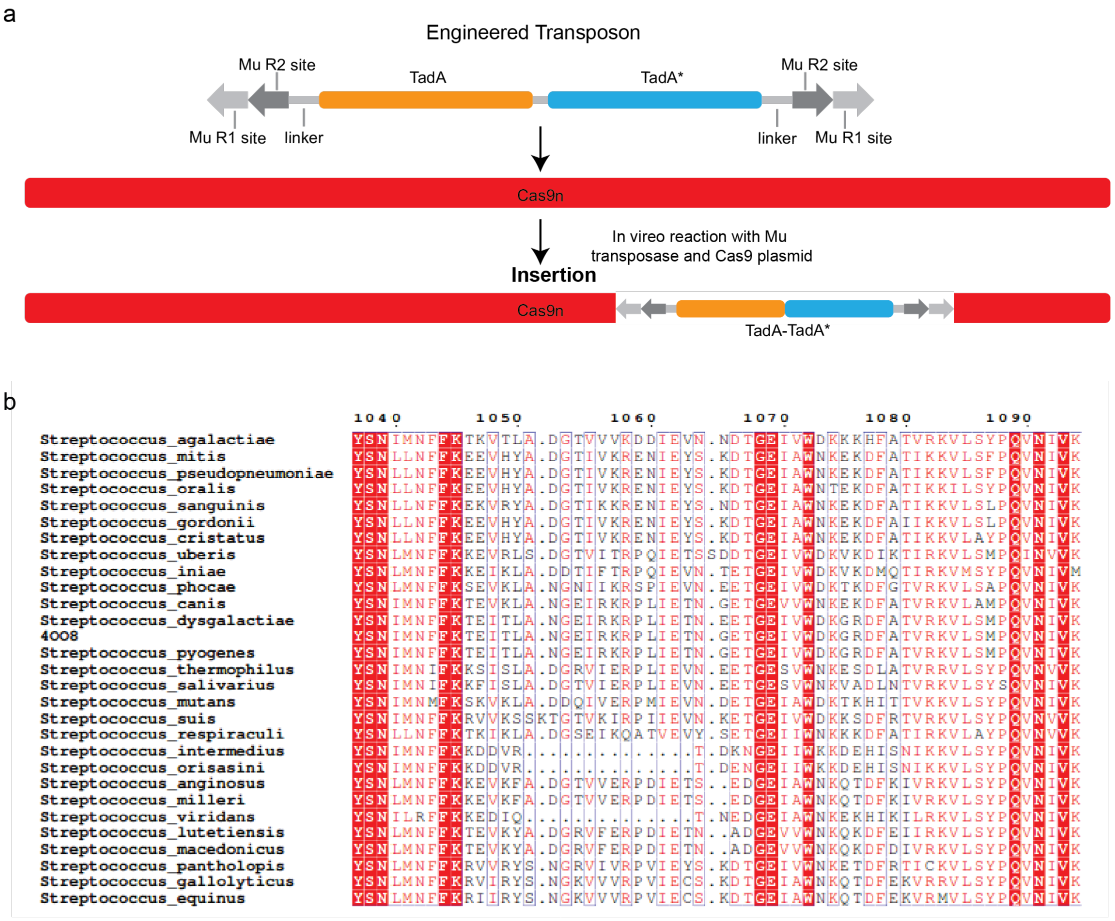

Supplementary Figure 1. Genetic screen for tolerant nCas9 sites. a, The transposon harboring TadA-TadA* and its random insertion into nCas9. **b,** Sequence alignment of SpCas9 (1036 to 1096) among 28 orthologs.

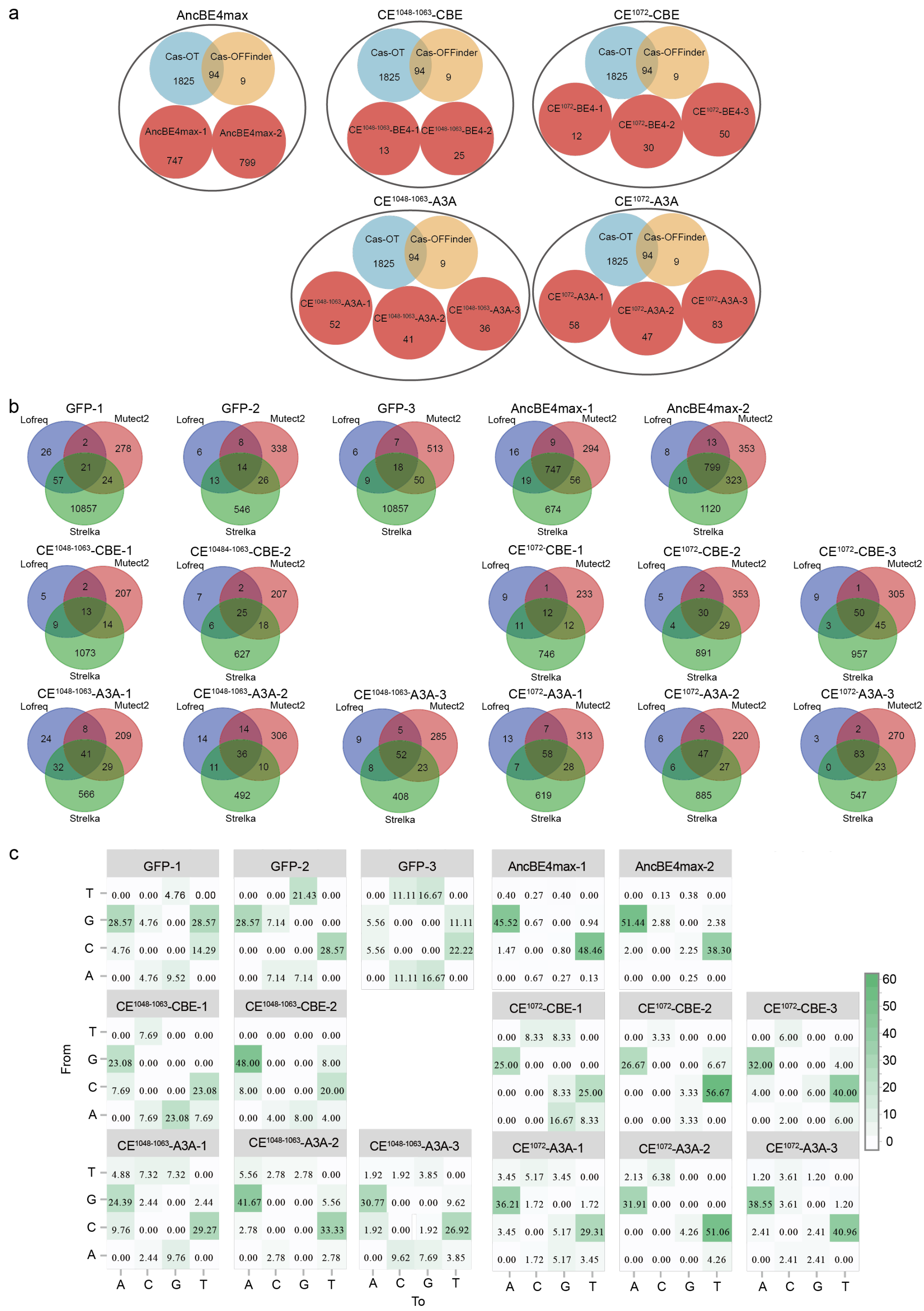

Supplementary Figure 2. GOTI data in detail. a, No overlap between the shared SNVs and the potential gRNA-dependent off-targets predicted by Cas-OT or OFFinder. **b,** SNVs in individual embryos treated with indicated proteins. Three software (Lofreg, Mutect2 and Strelka) were used to identify SNVs, with the shared SNVs detected by all the softwares considered genuine. **c,** Composition of the shared SNVs. The values are the frequencies of the indicated nucleotide alterations.
