# Supplementary material for: A Cas-embedding strategy for minimizing off-target effects of DNA base editors
Source: Nat Commun. 2020 Nov 27;11:6073. doi: 10.1038/s41467-020-19690-0 (PMC7695861; doi:10.1038/s41467-020-19690-0)
Supplement: Supplementary file 3 — Description of Additional Supplementary Files [file 41467_2020_19690_MOESM3_ESM.pdf]

Title: Supplementary Data 1:
Descriptions: Presents “Plasmid and primer sequences”

Title: Supplementary Data 2:
Descriptions: Presents “Mu-insertion library sequencing results”
