# Supplementary material for: A Cas-embedding strategy for minimizing off-target effects of DNA base editors
Source: Nat Commun. 2020 Nov 27;11:6073. doi: 10.1038/s41467-020-19690-0 (PMC7695861; doi:10.1038/s41467-020-19690-0)
Supplement: Supplementary file 6 — Reporting Summary [file 41467_2020_19690_MOESM6_ESM.pdf]

nature research

Corresponding author(s): Tian Chi

Last updated by author(s): Sep 25, 2020

# Reporting Summary

Nature Research wishes to improve the reproducibility of the work that we publish. This form provides structure for consistency and transparency in reporting. For further information on Nature Research policies, see our Editorial Policies and the Editorial Policy Checklist.

## Statistics

For all statistical analyses, confirm that the following items are present in the figure legend, table legend, main text, or Methods section.

| n/a | Confirmed | |
|---|---|---|
| ☐ | ☒ | The exact sample size (*n*) for each experimental group/condition, given as a discrete number and unit of measurement |
| ☐ | ☒ | A statement on whether measurements were taken from distinct samples or whether the same sample was measured repeatedly |
| ☐ | ☒ | The statistical test(s) used AND whether they are one- or two-sided<br>*Only common tests should be described solely by name; describe more complex techniques in the Methods section.* |
| ☒ | ☐ | A description of all covariates tested |
| ☒ | ☐ | A description of any assumptions or corrections, such as tests of normality and adjustment for multiple comparisons |
| ☐ | ☒ | A full description of the statistical parameters including central tendency (e.g. means) or other basic estimates (e.g. regression coefficient) AND variation (e.g. standard deviation) or associated estimates of uncertainty (e.g. confidence intervals) |
| ☐ | ☒ | For null hypothesis testing, the test statistic (e.g. *F*, *t*, *r*) with confidence intervals, effect sizes, degrees of freedom and *P* value noted<br>*Give P values as exact values whenever suitable.* |
| ☒ | ☐ | For Bayesian analysis, information on the choice of priors and Markov chain Monte Carlo settings |
| ☒ | ☐ | For hierarchical and complex designs, identification of the appropriate level for tests and full reporting of outcomes |
| ☒ | ☐ | Estimates of effect sizes (e.g. Cohen's *d*, Pearson's *r*), indicating how they were calculated |

*Our web collection on statistics for biologists contains articles on many of the points above.*

## Software and code

Policy information about availability of computer code

| | |
|---|---|
| Data collection | Illumina Hiseq , Illumina HiSeq X-Ten platform |
| Data analysis | EditR 1.0.8 , FlowJo, FastQC (v0.11.3), Trimmomatic (v0.36), STAR (v2.5.1), HaplotypeCaller (v4.1.2), Trimmomatic (v0.36), Sambamba (v0.6.7), BWA (v0.7.16), bam-readcount (v0.8), Mutect2 (v3.5), Lofreq (v2.1.2), Strelka (v2.7.1), CasOT (v1.0), Cas-OFFinder (v2.4). All softwares used in this manuscripts were described in the methods. |

For manuscripts utilizing custom algorithms or software that are central to the research but not yet described in published literature, software must be made available to editors and reviewers. We strongly encourage code deposition in a community repository (e.g. GitHub). See the Nature Research guidelines for submitting code & software for further information.

## Data

Policy information about availability of data

All manuscripts must include a data availability statement. This statement should provide the following information, where applicable:

- Accession codes, unique identifiers, or web links for publicly available datasets
- A list of figures that have associated raw data
- A description of any restrictions on data availability

High-throughput sequencing data are available in the NCBI Sequence Read Archive database under accession code (PRJNA660112).

# Field-specific reporting

Please select the one below that is the best fit for your research. If you are not sure, read the appropriate sections before making your selection.

- [x] Life sciences
- [ ] Behavioural & social sciences
- [ ] Ecological, evolutionary & environmental sciences

For a reference copy of the document with all sections, see nature.com/documents/nr-reporting-summary-flat.pdf

# Life sciences study design

All studies must disclose on these points even when the disclosure is negative.

| | |
|---|---|
| Sample size | Sample size is indicated on the Figure Legends. No statistical method was used to predetermine sample size. Sample size was chosen based on our previous works.(Zuo et al., Nature Methods, 2020; Zuo et al., Science, 2019) |
| Data exclusions | no data has been excluded. |
| Replication | All statistic data are presented as mean ± standard error of the mean. All samples represent a minimum of two replicates. The variation between different replicates is small, indicate all attempts at replication were successful. |
| Randomization | The samples were not randomized. Different cell passages were used for different biological replicates. Most of the target sites we choose to detect are pathogenic sites or reported in previous work (Wang et al., Nature Biotechnology, 2018). |
| Blinding | Blinding was not used. FACS and mouse embryo transplantation were used in the experiment. In order to ensure the rationality of public resources and the number of mice used, we did not use blinding. |

# Reporting for specific materials, systems and methods

We require information from authors about some types of materials, experimental systems and methods used in many studies. Here, indicate whether each material, system or method listed is relevant to your study. If you are not sure if a list item applies to your research, read the appropriate section before selecting a response.

## Materials & experimental systems

| n/a | Involved in the study | |
|---|---|---|
| x | | Antibodies |
| | x | Eukaryotic cell lines |
| x | | Palaeontology and archaeology |
| | x | Animals and other organisms |
| x | | Human research participants |
| x | | Clinical data |
| x | | Dual use research of concern |

## Methods

| n/a | Involved in the study | |
|---|---|---|
| x | | ChIP-seq |
| | x | Flow cytometry |
| x | | MRI-based neuroimaging |

## Eukaryotic cell lines

Policy information about cell lines

| | |
|---|---|
| Cell line source(s) | HEK293T (ATCC CRL-3216) and Neuro-2a (N2a) (ATCC HTB-96) |
| Authentication | Cell lines were authenticated by the supplier. 9 STR primer PCR the cell line then perform STR matching analysis. |
| Mycoplasma contamination | All cell lines tested negative for mycoplasma contamination. |
| Commonly misidentified lines (See ICLAC register) | None of the cell lines used was listed in the database of ICLAC. |

## Animals and other organisms

Policy information about studies involving animals; ARRIVE guidelines recommended for reporting animal research

| | |
|---|---|
| Laboratory animals | house mouse, C57BL/6J, female mice are 5weeks old, male mice are 8 weeks old. Mice were maintained in an Assessment and Accreditation of Laboratory Animal Care credited specific pathogen free facility under a 12 h dark-light cycle. Ambient temperature is 20 centigrade, relative humidity is 50%. |
| Wild animals | This study did not involve wild animals |

| | |
|---|---|
| Field-collected samples | This study did not involve field-collected samples. |
| Ethics oversight | All experiments involving mice were approved by the Animal Care and Use Committee pf the Institute of Neuroscience, Chinese Academy of Science, Shanghai, China. |

Note that full information on the approval of the study protocol must also be provided in the manuscript.

# Flow Cytometry

## Plots

Confirm that:

- [x] The axis labels state the marker and fluorochrome used (e.g. CD4-FITC).
- [x] The axis scales are clearly visible. Include numbers along axes only for bottom left plot of group (a 'group' is an analysis of identical markers).
- [ ] All plots are contour plots with outliers or pseudocolor plots.
- [x] A numerical value for number of cells or percentage (with statistics) is provided.

## Methodology

| | |
|---|---|
| Sample preparation | HEK293T and N2a cells were digested by trypsin (0.05%), contrifuged at 1000 rpm and filtered with a 35 µm nylon mesh. |
| Instrument | MoFlo XDP (Beckman) |
| Software | Summit Software version 5.2, FlowJo. |
| Cell population abundance | Cells transfected with plasmids were usually ~60% GFP+ and around 1,000,000 cells were sorted for RNA-seq. |
| Gating strategy | Positive and negative boundaries were determined by control cells that were not transfected with any plasmids. Cells with top 5% of GFP signal were collected. |

- [ ] Tick this box to confirm that a figure exemplifying the gating strategy is provided in the Supplementary Information.
